# Social prescribing from the patient's perspective: A literature review

**DOI:** 10.1002/jgf2.551

**Published:** 2022-04-26

**Authors:** Kazuo Araki, Yoshimitsu Takahashi, Hiroshi Okada, Takeo Nakayama

**Affiliations:** ^1^ Department of Health Informatics, School of Public Health Kyoto University Graduate School of Medicine Kyoto Prefecture Kyoto Japan

**Keywords:** coproduction, empowerment, patient's perspective, social prescribing, treatment evaluation

## Abstract

Social prescribing (SP) has aroused widespread interest across countries. SP is a way of linking patients in primary care with sources of support within the community by empowering patients to coproduce solutions to improve their health and well‐being. While previous research has demonstrated that SP contributes to reducing the total cost of the National Health Service, the analysis of its effects on patients is still inadequate. This literature review critically evaluated SP from the patient's perspective through the lens of medical anthropology. The review was made with respect to the three key concepts: treatment evaluation, coproduction, and empowerment. The study revealed that SP services in the UK enabled patients to feel comfort in many cases, but general practitioners, link workers, and patients should be collaborative with each other, and their interrelationships should not be hierarchical. Nevertheless, certain modifications may be needed to introduce SP in other healthcare systems.

## Graphical abstract

This article, based on a literature review, is the first evaluation of social prescribing from the patient's perspective through the lens of medical anthropology. The review revealed that social prescribing services in the UK enabled patients to feel comfort in many cases, but general practitioners, link workers and patients should be collaborative with each other, and their interrelationships should not be hierarchical.

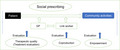

## 1 INTRODUCTION

In recent years, social prescribing (SP) has aroused widespread interest across countries, not only in the UK but also in Japan. SP, also known as “community referral,” provides a way of linking patients in primary care and their carers with nonmedical sources of support within the community.^1^, ^2^, ^3^ It is part of the National Health Service (NHS) Long‐Term Plan's commitment to make personalized care business as usual across the healthcare system.^4^ According to the Social Prescribing Network Conference,^5^ SP is a means of enabling general practitioners (GPs) and other frontline healthcare professionals to coproduce patients' prescription together with them so that they are empowered to find and design their own personalized solutions to improve their health and well‐being in the voluntary, community, and social enterprise (VCSE) sector. However, several different definitions of SP are already in use, as yet there is no standard or universally agreed definition.^6^, ^7^, ^8^, ^9^, ^10^, ^11^ In this study, SP is defined as a way of linking patients in primary care with sources of support within the community by empowering patients to coproduce solutions to improve their health and well‐being.

Since the 1990s, the biopsychosocial model of understanding health states and disease has emerged.^9^ In line with this, interventions focusing on the social components of care, such as SP, have increased. The increment of such interventions is partly because of the aging population, increases in chronic conditions, levels of social isolation, and growing burden of providing health care.^9^

According to Ogden,^12^ the three elements of SP are as follows: a prescriber—usually a GP but also a practice nurse or a healthcare assistant^10^; a (nonclinical) link worker—also termed a coordinator,^9^, ^13^ facilitator,^10^, ^14^ navigator,^15^ or referral agent/worker^16^; and a menu of activities. Examples of groups and services used by CHAT (the Community Health Advice Team established by Bradford South and West Primary Care Trust in 2005) include luncheon clubs, befriending groups, social services, volunteering organizations, getting back into workgroups, literacy classes, debt advice, access bus, bereavement groups, reminiscing groups, arts and craft groups, and music groups.^17^ The target audience of SP comprises the 20% of patients consulting their GPs for what is primarily a social problem,^5^, ^11^, ^18^ plus high users of hospital and GP services and those with the greatest health risks, such as diabetes or obesity.^12^

SP programs have been widely promoted and adopted in NHS as a means of dealing with some of the pressures on general practice. Several reports^1^, ^5^, ^19^ indicated that SP contributed to total NHS cost reductions. For example, various positive economic benefits to commissioners linked to the Rotherham Social Prescribing Pilot^1^ were confirmed, and the value of a range of social benefits associated with SP was estimated and confirmed, using financial proxies and techniques associated with social return on investment analysis.

On the contrary, Social Prescribing Network Conference identified five SP benefits in addition to cost‐effectiveness and sustainability: physical and emotional health and well‐being; behavioral change; capacity to build up the voluntary community; local resilience and cohesion; and tackling the social determinants of ill health.^5^ The following circumstances can be considered as the background of this.^7^, ^9^, ^19^ Since the 1990s, there has been a shift from the concept of the biomedical healthcare model to the biopsychosocial model for understanding health and disease, particularly for noncommunicable diseases. And in the last few years, interventions focusing on the social component of care have emerged, with some evidence for behavioral change. These aim to help people manage their chronic condition, prevent more serious health problems developing, and contribute to addressing health inequalities by building social support networks. The emergence of these interventions is in part because of the aging population, increases in chronic conditions, levels of social isolation, and growing burden of providing health care.^9^

In practicing in surgeries over time, GPs have gained awareness of problems that face their patients and communities and have appreciated that these problems are not purely medical or biological in origin. Therefore, Kimberlee, Moffatt et al., and Hutt stress on a holistic approach to manage long‐term health conditions.^6^, ^10^, ^20^ Thus, “GPs arguably have an opportunity to influence social and community networks, and this is the level at which social prescribing services tend to operate, […] making a link between GPs, patients and activities that aim to improve health and well‐being.”^20^ Those imply that GPs should take into consideration how social factors impact on patients' health, and therefore, action on health inequalities requires action across all the social determinants of health. Solutions to improve people's health and well‐being, especially in marginalized and underprivileged groups, are becoming increasingly popular in public policy because of greater stress on preventive interventions.

SP as one of such solutions focuses on secondary prevention by commissioning services that will prevent health deterioration in people with existing long‐term conditions and reduce costly medical interventions.^1^ In fact, SP has emerged as a useful tool for helping patients overcome some of the social and behavioral determinants of poor health.^21^ It is a way of linking patients in primary care with sources of support within the community to help improve their health and well‐being.^8^, ^21^ Thus, SP is tailor‐made for VCSE‐led interventions and is expected to result in: better social and clinical outcomes for people with long‐term conditions and their carers; more cost‐efficient and cost‐effective use of NHS and social care resources; and a wider, more diverse, and responsive local provider base.^1^, ^4^

Bickerdike et al. carried out a systematic review^8^ that appeared to be a turning point in research on SP. The authors assessed the evidence for the effectiveness of SP, based on nine databases for the period from 2000 to January 2016 in the UK. Despite clear methodological shortcomings, most evaluations presented positive conclusions. According to the authors, the current evidence lacks sufficient detail to judge either success or value for money, and if SP is to realize its potential, future evaluation must be comparative by design and consider when, by whom, for whom, how well, and at what cost. Other more recent systematic reviews^3^, ^16^, ^22^, ^23^ have revealed that the evidence base for SP remains considerably behind practice, and well‐conducted research and transparent reporting of findings are required to improve the evidence base. Thus, because of a weak evidence base for SP services in addition to little consensus on appropriate outcome measures,^24^ most authors have agreed that more research is needed on the benefits to patients and professionals.^10^, ^16^, ^17^, ^18^, ^20^, ^21^

In brief, previous research lacks both appropriate criteria/clues and broad perspectives beyond medical science for the evaluation of SP. Therefore, this literature review aimed to critically evaluate SP from the patient's viewpoint through the lens of medical anthropology, by referring to a couple of key concepts as clues for the evaluation.

## 2 METHODS

### 2.1 Setting

SP services in the United Kingdom.

### 2.2 Key concepts

Given this study's focus on the experiences and views of SP service users, that is, patients, the study drew on qualitative and mixed‐methods analyses of SP, including narratives mainly of patients, as well as on social science literature considered relevant to SP.

As defined previously, SP refers to a way of linking patients in primary care with sources of support within the community by “empowering” patients to “coproduce” solutions to “improve” their health and well‐being.

A prerequisite for evaluating SP (and treatment in general) is from what perspective it is evaluated (e.g., improved or not). In other words, how SP should be evaluated is to be clarified in the first instance. There are various approaches to treatment evaluation, namely those from the patient, the practitioner, the social scientist, and the natural scientist.^25^, ^26^ Therefore, “treatment evaluation” is considered a key concept in the present study. “Coproduction” and “empowerment” should also be key concepts in SP, as those are included not only in our definition but also in the definition made or cited by other authors.^5^, ^11^

Thus, the key concepts to be used as clues for the evaluation of SP in this study are “treatment evaluation,” “coproduction,” and “empowerment.”

### 2.3 Literature search

Qualitative and mixed‐methods analyses and systematic and literature reviews relating to SP and gray literature relevant to SP and to the above‐mentioned key concepts were searched for—one after another, starting from the publication^4^ of NHS—in PubMed, SOLO (Search Oxford Libraries Online), and Google Scholar.

### 2.4 Defining the key concepts

Each of the key concepts was defined and considered for its relevance to SP.

### 2.5 Analyzing narratives

For each of the key concepts, the narratives of mainly patients were analyzed and classified into several groups, because those narratives provided access to individual patients' illness experience.^27^, ^28^

### 2.6 Evaluating SP

The evaluation of SP from the patient's perspective based on the narratives was performed in connection with the key concepts.

## 3 RESULTS

### 3.1 Treatment evaluation

#### 3.1.1 The concept and its relevance to SP

The study focused on the impacts of SP on patients. In other words, whether SP interventions were successful from the patient's perspective, how, and to what extent were examined.

Donabedian classified approaches to evaluation of the quality of care into three headings: structure, process, and outcome.^29^ The treatment evaluation from the patient's perspective is related to all these aspects.

Lock's study of menopause in Japan and North America addressed the Cartesian body affected by either disease or illness.^30^ Today's medical anthropologists (e.g., Farquhar and Lock^31^) are looking for ways in which to consider the social in the biological and vice versa. This should also apply to treatment evaluation.^26^

There are different approaches toward treatment evaluation. Some of those methods are explicitly directed at evaluating the quality of the treatment from the patient's viewpoint (“therapeutic quality”), whereas others record the practitioners' perceptions, their conceptualization of patient's condition, their calculations, and the results of their intervention (“therapeutic results”).^25^ Furthermore, the distinction between socially constructed illnesses^32^ and biological dysfunctions of disease^33^ leads to differentiating the social scientist's assessment of “therapeutic success” from the natural scientist's “therapeutic efficacy.”^25^

Csordas^34^, ^35^ combined Merleau‐Ponty's understanding^36^ of the body as the existential ground for human beings to experience the world with Bourdieu's practice theory,^37^ which postulated that even the body we might experience as primordial and natural was ultimately derived from social practice and was thereby informed. This approach, which suggests that the social is intertwined with the natural in a complex way, promises to provide a rich theoretical framework for treatment evaluation.^26^ The way a patient feels after treatment ultimately determines the impact of the treatment on the patient's self and body, on feelings and subjectivity, or on intersubjectivities between a patient and a practitioner, a patient and other patients, patients and their social and natural environment, and patients and the material things they interact with.^26^ “Intersubjectivity,” coined by Husserl,^38^, ^39^, ^40^ can be stated as the interchange of thoughts and feelings, both conscious and unconscious, between two (or more) persons or subjects, as facilitated by empathy.^38^, ^39^ What is problematic is, however, that feelings are difficult to pin down and define, because “[t]hey are experienced in response to the situation in its entirety, in which the treatment is administered.”^26^ It is often impossible to distinguish between those that caused the treatment itself and those that were primarily affected by the environment. In general, feelings are fluid and difficult to label. They are an aspect of interpersonal relationships.^41^ “What one feels after a treatment is often what one was told to expect before deciding to have the treatment.”^26^

Moreover, the safety that people feel is told to determine the social, psychological, and occupational benefits that are enjoyed.^42^ On the patient's side, the subjective experience of feeling safer or lighter appears to be pervasive. People speak of “feeling lighter” as a sign of betterment cross‐culturally in different languages.^26^ Thus, effective treatment makes patients experience their body and self as “feeling lighter.”

Furthermore, there is more to be learnt from what is said about ritual process composed of three stages: predisposition, empowerment, and transformation.^43^, ^44^, ^45^ These stages are essential for making possible the all‐compassing bodily, emotional, and mental transformations that make treatment effective.^43^

In view of the above, the concept of “therapeutic quality” from the patient's perspective is considered more appropriate for this study on SP from the patient's perspective than the three others. It should be noted, however, that even the treatment evaluation based on “therapeutic quality” is highly dependent on the circumstances where the patient is placed.

#### 3.1.2 Narratives

The narratives relating to treatment evaluation were classified into seven categories: “recognition of the present situation,” “a sense of purpose,” “being with others,” “feeling lighter,” “threshold moments,” “moving forwards,” and “need for improvement” (Table 1).^9^, ^13^, ^42^, ^46^, ^47^, ^48^, ^49^

**Table 1:** Narratives relating to treatment evaluation

**Recognition of the present situation**
Recognition of the present situation is a starting point for everyone. Carnes et al. carried out a mixed‐methods evaluation of the impact of a SP service on patients in primary care, using patient surveys with matched control groups and a qualitative interview study.^9^ The quantitative study indicated that SP did not have any statistically significant effects on patients' general and mental health, well‐being, and active living changes. On the contrary, the qualitative study showed that most patients had a positive experience with SP (“therapeutic quality”). For example, some of the most positive outcomes reported by patients resulted from experiencing sessions, which allowed them the time to explore their situation more fully and work collaboratively to set realistic goals for the future (Narratives 1–3).^9^ The reason for the discrepancy between the results of the quantitative and qualitative studies is not clear but, as Carnes et al. mentioned, might be a statistical one because of the large number of controls.^9^ Thus, they concluded, “Our qualitative study elicited strong positive narratives similar to case studies reported in other evaluations […] but the quantitative data did not support or reflect the strength of these narratives throughout the whole referred group.”^9^
It's done me a world of good, taken me out of the house, given me a routine and given me a sense of purpose and …hope. It's given me back my confidence. (Practitioner engaged)^9^ It [SP] gave me the motivation to think I might be ready to go back to work. (Practitioner engaged)^9^ It [a voluntary organisation return to work scheme] allowed me to keep my hand in, so when I was ready to go back to work [this meant] I wouldn’t have not been working since 2012…I’ve [now] got references and skills that are current. (Practitioner engaged)^9^
**A sense of purpose**
Hassan et al. noticed that the most significant aspect of the Life Rooms (a SP service run by a NHS foundation trust) was the approachability of the Life Rooms members (staff, volunteers, and other service users) and their understanding of participants' needs (Narratives 1–3).^46^ Woodall et al., whose quantitative analysis revealed positive outcomes, reported that accessing a range of activities such as swimming and “hobby” activities provided a greater sense of independence and gave individuals a sense of purpose (Narrative 4).^47^
4 It's just such a safe place because, even if I am not in a good mood, I get out and at least go to the Life Rooms where I know other members of staff and students and service users…everybody understands. (FGD6_SU)^46^ 5 …she spoke to me about all my problems and how I was getting on. All very informal and I can cope with that, but I couldn't cope with talking to a doctor looking at the time all the time. (FGD5_SU)^46^ 6 They don't treat you like a patient, they treat you like you're just a person and I think that makes a big difference. (FGD2_SU)^46^ 7 I’d been feeling very depressed, I’ve been in the building trade for fifty years very active, doing all my own repairs at home I was a joiner. And then I’m suddenly stuck in a wheelchair. And it was more frustration. In my mind I could still do the job but physically I couldn’t. And everything was load onto my wife. You know she was having to do things that I used to do I had to sit and watch her …and it just got me down. Still does at times… the service just gave me suggestions on things to do like one thing I’ve always enjoyed is swimming. And I haven’t done it for years. And it was you know accessing things like that. There is a workshop where people go to do wood work…I feel a bit better in myself knowing that there are things out there that I can do. (Male client: interview 12, aged 50 years and over. Referred to the social prescribing service by GP)^47^
**Being with others**
There could be multiple reasons why many people being involved in SP felt comfortable. According to Redmond et al., the opportunity to “be with others” was the most common response given by the participants in “Artlift courses” (creative arts courses) as a form of SP (Narratives 1–4).^48^ The importance of “being with others” that could be, in these cases, termed “intersubjectivity” was also confirmed by Bertotti et al. (Narrative 5)^13^ and Stickley and Hui (Narrative 6).^42^ Hassan et al. pointed out that the Life Rooms enhanced social inclusion and connectedness and were described as social hubs for the majority of participants (Narrative 7).^46^
8 Meeting people. Also finding out what I can do! (Respondent 4027)^48^ 9 I enjoyed being with other people and doing painting. (Respondent WS006)^48^ 10 Learning something new, being in a group. (Respondent 0271)^48^ 11 Interaction with like‐minded people. (Respondent 3133)^48^ 12 Best thing has been meeting new people and making friends. My mobile full up with names and numbers of friends before it was just family and doctor's number. (Service user)^13^ 13 … for me to sit in a group, it's, it is incredible for me, and then now I feel I can go and do an art course, and get on with it… (Rhianon)^42^ 14 I met someone there and we clicked and we had lunch in the little coffee shop there and it was like oh my god this is the first time in my adult life I have sat and had lunch with a friend. (FGD6_SU)^46^
**Feeling lighter**
Having the opportunity to attend support groups in the local community, facilitated through the SP service, enabled patients to gain more of a balanced perspective by being able to share experiences with others going through similar difficulties. This resulted in some individuals' “feeling much lighter” or more hopeful about their own lives (Narrative 1).^47^
15 Since I went to that group, I could see what other people are actually having difficulty in life with, and you do not assess yourself the same. It actually made me realise that life is not all about yourself. You find here that everybody has got different problems. You find that yours is not even as serious as the other person that you are talking to. (Female client: interview 2, aged under 50 years. Self‐referred to the social prescribing service)^47^
**Threshold moments**
Redmond et al. discovered also other thematic areas in the responses by the participants than “being with others.”^48^ Some participants expressed the view that what they had enjoyed most about the Artlift courses was concerned with what they had escaped from the everyday (e.g., Respondent B0237; Respondent B0146; Respondent B0417). Moreover, Artlift provided an opportunity for respondents to take, for example, “…time for myself…” (Respondent B0202) and “…learn new skills…” (Respondent 1559). Furthermore, many other respondents found the Artlift sessions on offer to be a reward in their own right: “…the opportunity and encouragement to try something I've never done since childhood…” (Respondent B0490). The Artlift activities provided a sense of respite: “time out” (Respondent B0022), “[t]ook my mind off my pain” (Respondent A1524), “mind distraction [that] helps depression” (Respondent 1480), “having something to keep my hands busy” (Respondent 4081), and “switching off” (Respondent B0057). The study by Redmond et al. revealed also that Artlift provided opportunities whereby participants were able to encounter, identify, and begin to document “threshold moments,” in which they were able to recognize personal growth and change, and then progress with a change in their psychological outlook (Narratives 1–3).^48^ Being moved out of a dark place located these participants elsewhere; specifically, they implied they were no longer in the place they had occupied prior to starting Artlift.^48^
16 I found the session cathartic. (Respondent 1483)^48^ 17 It's moved me on. (Respondent B0259)^48^ 18 I have been in a dark place… (Respondent A1798)^48^
**Moving forwards**
Service users felt that there were significant changes to their daily lives (Narrative 1).^49^ Participants in the Life Rooms described a journey of self‐development, developing new hobbies and interests, enhancing confidence, and gaining independence (Narratives 2–5).^46^
19 I built up so much energy, I'm getting back to what I like doing and I'm moving forwards going into doing my other volunteer job later in the year. And I am meeting all sorts of new people and it's great, you know. (Service user 4)^49^ 20 I am out of bed, I am dressed, depending on the day whether I'm able to cope with the shower but certainly washed, dressed and today I am out the house, I am here. (FGD1_SU)^46^ 21 I am able to go in and actually do my shopping instead of having rely on people, getting my independence back is huge for me. (FGD4_SU)^46^ 22 I would not have been able to do it if I hadn't have gone to Life Rooms, it give me coping mechanisms, it's given me strategies, and its helped me to get the confidence and self‐esteem because I was at rock bottom. (FGD1_SU)^46^ 23 I am now seeing things differently about myself. Since doing these courses I understand my illness more and I understand if I am having a bad day. It's also helped me be able to voice things better as well. I can tell people more about my mental health…Even if it's the middle of the night and I'm struggling, I know that there are people that I can phone and just say “I am not feeling great.” So that's how it's helped. (FGD4_SU)^46^
**Need for improvement**
The above statements do not mean, however, that the patients made best use of the SP service system to its full extent and were totally satisfied with it.^9^ According to Woodall et al., some interviewees stated that it would be useful to have a greater number of one‐to‐one sessions should they need to (Narratives 1–2).^47^ In this respect, Husk et al. pointed out that, for SP interventions to be successful, patients should be successfully transferred from the primary care setting to the relevant resource and to maintain participation for an appropriate period of time.^7^ The frequency of contact with link workers varies from patient to patient, depending on need and circumstances, and contacts can be face‐to‐face, via telephone, email, etc.^10^ To avoid dependency on the SP service, individuals are encouraged to “exit” the service or are referred to other health and social care providers after six sessions; most clients receiving appointments exit the service within 16 weeks, with the mean length of time being ten weeks.^47^
24 I think it probably could have been longer. I think it should be more like help until they think they are done. Cos when I first met her [Wellbeing Coordinator] I was really down, but towards the end I was much better but I still could have done with one or two more. (Male client: interview 4, aged under 50 years. Referred to the social prescribing service by GP)^47^ 25 The time wasn't really enough. I wish the time was a bit extended. I requested more time. It's not enough time to sort out everything that a person would actually want to do. What I wanted we couldn’t really sort out everything. (Female client: interview 2, aged under 50 years. Self‐referred to the social prescribing service)^47^

### 3.2 Coproduction

#### 3.2.1 The concept and its relevance to SP

SP is not just a means of referring patients to sources of support in the community through healthcare professionals but that of helping patients “coproduce” solutions to improve their health and well‐being. Realpe and Wallace state that coproduction refers to the contribution of service users to the provision of services.^50^ Needham and Carr describe coproduction as a collaborative relationship between the people who use services and the formal service provider,^51^ although it is not a panacea.^52^ In this study, coproduction is defined as a way of working whereby service users and providers work together to create decisions or services, which work for them all.

Coproduction is applied to the collaboration between a professional provider and a service user. It implies a change in the role of professionals from fixers of problems to facilitators working with their clients to find solutions, whereas patients need to transform themselves from passive to active users of SP with the cooperation of healthcare professionals and link workers. At the same time, it involves the “empowerment” (as defined later) of frontline staff in their everyday dealings with customers.^51^

Furthermore, Social Care Institute for Excellence points out that, in order to put coproduction into practice, four factors are essential: “culture”—the beliefs and values that define an organization and its way of working; “structure”—how the organization is arranged and the systems it has set up to perform its work; “practice”—how the organization and the people who work for it carry out their work; and “review”—monitoring how the work is performed and the outcomes or impacts of the work.^53^ Furthermore, Carnes et al. identified two overarching themes from the data obtained in their mixed‐methods evaluation of a SP service: “processes and procedures” and “engagement and outcomes.”^9^ The former appears to correspond to “culture” and “structure,” and the latter to “practice” and “review.”^53^ As getting patients involved in the whole process of SP appears necessary, this sort of grouping is considered relevant to discussions about SP. Thus, coproduction is a concept closely related to SP and could be a clue for its evaluation.

#### 3.2.2 Narratives

The narratives relating to coproduction were classified into two categories: “processes and procedures” and “engagement and outcomes” (Table 2).^9^, ^13^, ^15^, ^46^, ^47^, ^54^

**Table 2:** Narratives relating to coproduction

**Processes and procedures**
According to Hassan et al., participants spoke about the Life Rooms setting that facilitated easy access to many resources without additional costs and administrative burden (Narratives 1, 2).^46^ Carnes et al. noticed that a patient was pressed for time; that is, he or she had no time for thinking of SP (Narrative 3).^9^ This situation could be resulted from the fact that some interviewees were not sure what SP was and who the service delivery organization was, despite having been referred to (Narrative 4).^9^ This may be, in addition to the lack or insufficiency of culture and structure,^53^ because the people interviewed met so many different healthcare professionals that they had lost track of who they were seeing (Narratives 5, 6).^9^ A similar situation was also observed by Bertotti et al. (Narrative 7).^13^ Both service users and providers discussed the coconstructed and consultative nature of the service. Rather than being dictated to, patients felt assured that the process was very much about working together to decide on the best course of action (Narrative 8).^47^ An interview with a patient conducted by Bertotti et al. also confirmed the importance of giving the opportunity for patients to get involved in the process to have face‐to‐face sessions with SP coordinators (link workers) (Narrative 9).^13^
[in other services] you would have to fill in loads of forms or you would have to apply online or you would have to get some type of funding, but since I have been here, I have done loads of stuff and I have never been asked for a penny. (FGD3_SU)^46^ It could be is just me coming in and saying to (name) have you got time for a cup of coffee and a chat for ten minutes. That sort of conversation will sort me out if I was feeling a bit rough in the head, a bit fed up, just somebody talking to me like that, it doesn't have to be sitting with a counsellor. (FGD1_SU)^46^ I had too many other things going on [family crises]. (Practitioner not engaged)^9^ I have no idea who or what you are talking about, but sounds a good idea, I don’t know why I was referred… (Practitioner not engaged)^9^ I don’t know who she was [in terms of health care professional]…I can’t remember her name…errr but she was very nice. (Practitioner engaged)^9^ The problem is there are lots of services and lots of names, I get confused.’ (Practitioner partially engaged)^9^ I have no idea who or what you are talking about, but sounds a good idea, I don’t know why I was referred… (service user)^13^ I was free at any time to say ‘no I’m not comfortable with this I don’t like it’ and she [Wellbeing Coordinator] was very adamant that it would not affect me if she’d arranged it all and I’d have gone and then come back and said ‘no I can’t do this’ she’d have been fine with that. It was kind of all along how I felt and she made that very clear that any time that I didn’t feel comfortable with anything that she maybe suggested or got me to have a look at, if I didn’t like the idea it was no problem. (Female client: interview 3, aged 50 years and over. Referred to the social prescribing service by GP)^47^ You feel able to offload if you need to, discuss your fears – it's about not being so hard on myself and validating myself. (service user)^13^
**Engagement and outcomes**
With respect to “engagement and outcomes”^9^ or “practice and review,”^53^ some people did not even believe in the first instance that they or their family members needed SP and benefitted from it (Narratives 1–6).^15^, ^46^, ^54^ For this reason, as Woodall et al. noticed, getting patients involved in the engagement in cooperation with link workers is important, since link workers (LW) are more empowered^50^, ^51^ (Narratives 7, 8).^47^ This kind of “coproduction” is also underpinned by Pescheny, Randhawa, and Pappas (Narrative 9).^15^
10 [The LW] said that both of us could go to [the group] the first time, so that she could help me make sure I was comfortable and that I had what I needed to do the class. She spoke to [the instructor] and introduced me to her. I felt a lot happier knowing I had someone I knew to go with me. [lines omitted] If someone had just told me to go, I don't think I would have gone. (Patient 8)^54^ 11 You're kind of helping each other, because I think for most people [with this condition] you kind of feel that you're the only person on the whole of Plant Earth, you know. You don't seem to know how many other people [have this condition] so the fact that you can meet up with others is like, oh, there are other people that understand and know how it's difficult (…) and so, you were able to give each other encouragement or copy each other or learn from each other. (Patient 4)^54^ 12 So, I didn't know there were people out there like me, and [LW] made me realise (…), there are lots of people out there like me and we're like a little tribe. And there's little places we can go and hook up and just kind of like talk about anything you want, or not talk at all. And I just think it saved me. Honestly, I don't know what would have happened. It terrifies me to think what would have happened. I think I would have got more ill, if I'm honest, because I was desperate. (Patient 3)^54^ 13 You can come here for say 10 minutes, 20 minutes, half an hour and just those few minutes or second or that bit of time you spend with somebody here who's nice to you can make you feel a bit better but you are in charge of what you are doing. I think it's really, really important and just that little bit of control can you make feel on top of the world, you can go away thinking I did something really good today. (FGD1_SU)^46^ 14 They give you a bit of a plan and they can help you along your way and they will always support you, if you come back and you go that did not work or I am having problems with this they can support you. (FGD5_SU)^46^ 15 I had a person who declined […] she was a carer for a [man with dementia], she goes like: ‘Actually I have things to do, they take me out’. She actually felt that she did not need to get involved because she is already doing enough other things and getting support from other areas, so she did not feel the need. (GP1)^15^ 16 And to be taken notice of. And to be looked on as a person as an individual as opposed to ‘oh just somebody else’. (Female client: interview 9, aged under 50 years. Referred to the social prescribing service by GP)^47^ 17 It's that being able to talk to somebody, and somebody being willing to listen, I think that's the crux of it, and not being judgmental. (Male client: interview 12, aged under 50 years. Referred to the social prescribing service by GP)^47^ 18 And I said that I also want something sort of that occupies my mind. And that is when she [social prescribing navigator] suggested the art class, which has been absolutely brilliant and exactly what I wanted […] and I explained on the physical side that I am severely limited. She printed out for me the gym programmes at the various health centres, so I could decide where I wanted to go. (Service user 3)^15^

### 3.3 Empowerment

#### 3.3.1 The concept and its relevance to SP

“Empowering people, citizens, consumers, and patients is critical for improving health outcomes, health system performance, and patient satisfaction.”^55^ Within the framework of SP, patients are expected to be empowered to coproduce solutions in the hope of improving their health and well‐being. Empowerment is, as mentioned earlier, one of the three stages of a ritual process.^43^ Defining empowerment is, however, not an easy task. The fundamental question is whether empowerment is being considered as a process or an outcome.^56^ In fact, according to McAllister et al.,^57^ there are many definitions, with most relating in some way to patients conceived as self‐determining agents with some control over their own health and health care, rather than as passive recipients of health care.^56^, ^57^, ^58^, ^59^, ^60^ Those definitions focus on individuals' capacity to make decisions about their health/health behavior and to take control over aspects of their lives that relate to health. In this connection, it should be reminded that, in the empowerment relationship, there needs to be decision making, not only by frontline staff including healthcare professionals but also by patients who are less empowered.^51^ All in all, as Weiner stated, the word “empowerment” is a fitting term for the “process” by which people gain—at least to a certain extent—mastery over their own affairs.^61^

Bond and Csordas dealt with powerlessness and empowerment in their article to question how women in Alcoholics Anonymous (AA), a support group for alcoholism, navigated and negotiated the contradictions found within a male‐dominated and male‐centered program.^62^ The authors revealed that empowerment resulted from admitting powerlessness (paradox of powerlessness).^62^ The patients referred to SP could be similar, as to their “weak” situation in a sense, to the alcoholic women in AA.

What, then, is power? Foucault stated that the effects of power could be either positive or negative.^63^ What is relevant for us is how power is engaged and how relations of power are exerted in practice. According to Bond and Csordas, the women interviewed gained power through an organization that was critiqued as a source of negative power over women by engaging it as a productive power with/to change their lives.^62^ In clinical settings where paternalism has traditionally been prevalent and therefore patients have had little power, a new paradigm would have to be commonly put into practice: the shared decision‐making approach. Thus, power should, in principle, be interpreted positively for the purpose of this study.

As demonstrated previously, therapeutic or, more generally, healthcare evaluation relies on various factors including, among others, the perspectives applied. Healthcare evaluation has recently been required to take the perceived value of nonhealth outcomes such as empowerment, a psychosocial outcome, into account.^57^ According to McAllister et al.,^57^ the separation of health status from psychosocial outcomes was first advocated in chronic disease by Kleinman^27^; since then, patient empowerment has gained credibility in health care, reflecting moves away from paternalistic models toward more equitable/collaborative models of clinician‐patient interaction, including shared decision making. The shared decision‐making approach is a product of reflection on the conventional evidence‐based medicine (EBM), as the evidence alone is never sufficient to make a clinical decision.^64^ It is an approach contrary to the paternalistic one. Shared decision making has indeed become popular in clinical settings in recent years. In this approach, both patients and clinicians bring evidence, values, and preferences to the decision,^64^ thus empowering patients to participate in clinical decisions and seeking to ensure decisions consistent with patient values and preferences.

In the present study, based on the previous studies cited above, empowerment is defined as a process of increasing the capacity of patients to make decisions and transform those decisions into effective actions and outcomes. Therefore, it could be a clue for evaluating SP.

#### 3.3.2 Narratives

The narratives relating to empowerment were classified into two categories: “paternalism and some empowerment” and “empowered by someone more empowered” (Table 3).^9^, ^10^, ^13^, ^15^

**Table 3:** Narratives relating to empowerment

**Paternalism and some empowerment**
Narratives related to empowerment were few. Nevertheless, some of them indicated that paternalism was still pervasive (Narrative 1),^15^ and in this case, there was virtually no “decisional control.”^57^ Carnes et al. also revealed a similar situation (Narrative 2).^9^ In other interviews with patients carried out by Pescheny et al., empowerment was also observable—to a certain extent—in the sense that the decision in favor of SP (e.g., in contrast to medication) is left to the patient's judgment (Narratives 3, 4).^15^ In most cases, the patients referred to SP appeared to have made judgments, “hoping for the future.”^57^
If, I mean, even if possibly another doctor would have recommended it, the thing is I know [name of GP]. We know each other for so long, I trust him. And I trust him that he knows me well enough, so I said ‘yeah okay’. (Service user 3)^15^ My GP knows me so well he probably just referred me because he thought it would be good for me. (Practitioner partially engaged)^9^ One question was: ‘Do I need help?’ And my answer was: ‘Definitely yes!’ And it all sort of started from there really. (Service user 2)^15^ Well, I do not want to pop any more pills than I have to. I regard pills as a short‐term solution and I thought this [social prescribing] is something more than a short‐term solution. I mean happy pills might get me through the winter, but what then? (Service user 3)^15^
**Empowered by someone more empowered**
Face‐to‐face consultation between a patient, on the one hand, and a link worker (SP coordinator) who is more empowered, on the other,^50^, ^51^ provides the patient with necessary information, builds trust between them, generates an opportunity for the patient to be empowered to explore his or her needs and aspirations, and coproduces solutions for his or her benefit. Thus, support by a link worker appeared indispensable to allow patients to be empowered to make decisions, as no one would be able to be empowered by another person himself or herself not empowered (Narratives 1, 2).^10^, ^13^
5 I just expected the Link Worker to introduce me to the gym, and that would have been it. And I think, if it had just been [that] I would have turned round, and I would have gone the opposite direction. But because of the way it was so gradually and really professionally linked in to different things, I just felt as though I'd floated into it, rather than getting shoved from behind. I just felt as though I was gradually moved into it. (P2, female, 70–74 years)^10^ 6 It would have been much nicer if they [social prescribing coordinators] had had a conversation face‐to‐face cause it felt like I was sitting there and they were at the desk trying to write everything down quickly…I think a better way would be someone is giving you eye‐contact rather than just writing things down and you’re thinking what are they writing? (service user)^13^

## 4 DISCUSSION

The preceding section provides evidence about the impact of SP on health and well‐being of patients. This study is unique, showing insight into outcomes of SP services from a broad perspective beyond medical science. Furthermore, the study indicated that the concepts of “treatment evaluation,” “coproduction,” and “empowerment” were useful clues for the evaluation of SP from the patient's viewpoint. In the following, the results in the previous section will be synthesized and evaluated by referring to these key concepts.

As regards “treatment evaluation,” one of the most positive outcomes reported by the patients resulted from experiencing sessions, which allowed them the time to explore their situation more fully and work collaboratively to set realistic goals for the future. Accessing a range of activities such as hobby activities provided a greater sense of independence and gave individuals a sense of purpose. Other narratives showed that SP services enabled patients to gain more of a balanced perspective by being able to share experiences with others going through similar difficulties. The opportunity of “being with others” or intersubjectivity in a sense was the common response given by the patients involved. Such opportunity resulted in some individuals' “feeling lighter” or more hopeful about their own lives. There could be multiple reasons why many people being involved in SP felt comfort. This kind of experience provided “threshold moments” and “moving forwards,” in which they were able to recognize personal growth and change. All in all, most patients referred to SP evaluated it positively from their own perspective (“therapeutic quality”). However, some patients claimed that there was some room for improvement in the SP scheme, and a holistic approach should be adopted to make better use of SP services.

In connection with “coproduction” and “empowerment,” reference is often made to “person‐centered/person‐centeredness.” The term “person‐centeredness” is a broad concept. It is, in fact, to ensure that people's preferences, needs, and values guide clinical decisions and, thus, to provide care that is respectful of and responsive to them. This goal could be reached through coproduction of health and well‐being outcomes by patients, GPs, and link workers working in collaboration, that is, by empowering patients to some extent. Thus, person‐centeredness mainly includes coproduction and empowerment. Therefore, we have not explicitly included person‐centeredness in our key concepts in addition to coproduction and empowerment.

With respect to “processes and procedures” of “coproduction,” the narratives revealed that some patients had virtually no time to think of SP and lacked in knowledge about it. There was confusion among patients because so many professionals were involved. Nevertheless, patients felt assured by working together with practitioners to decide on the best course of action rather than by being dictated to. Patient narratives also confirmed the importance of having the opportunity to get involved in the process to have face‐to‐face sessions with link workers. As regards “engagement and outcomes,” some people did not even believe that they or their family members needed SP and benefitted from it, although several narratives indicated that getting patients involved in the engagement in cooperation with link workers was important, as link workers were more experienced and had more power than patients. To sum up, patients appeared to consider coproduction to be important for their own benefit but were involved in coproduction to a limited extent.

Narratives indicated that “paternalism” was pervasive and, in this case, there was virtually no “empowerment” in the sense of decisional control. However, empowerment was observable—to a certain extent—in the sense that the decision in favor of SP (e.g., in contrast to drugs being prescribed by GPs) was left to the patient's judgment. Nevertheless, the patients referred to SP appeared to hope for the future. Patients recognized that they should be “empowered by more empowered people” such as GPs and link workers. And, because of the asymmetry of information, involvement of such professionals was considered indispensable in most cases. To summarize, patient empowerment did not appear to have been commonly put into practice, probably because the awareness of SP among patients was insufficient and paternalism was still prevalent.

To conclude, this study suggests that SP services enable patients as service users to feel comfort, thus leading to a more positive and optimistic view of their life, often through offering opportunities to engage in a wide range of activities. SP schemes could enhance individuals' awareness of their own health, and this may uncover further needs that may require primary care intervention, although SP services have also been sought to reduce healthcare utilization, particularly GP and primary care services. To offer SP services, it is necessary to recognize and evaluate the needs of potential service users. Otherwise, no SP services tailored to the specific needs of service users would be able to be offered, even if there are abundant resources on hand. Therefore, the roles of both GPs as first contacts and link workers as intermediaries are of primary importance. The deep involvement of link workers and GPs remains essential. However, when focusing on the patient's perspective, GPs, link workers, and service users should be collaborative with each other, and their interrelationships should not be hierarchical. SP offers ways to address some of the broader determinants of health and extends the boundaries of primary care for both patients and healthcare professionals. It also provides a mechanism to bridge the gap between primary care and the VCSE sector to deliver support tailored to individual needs. Nevertheless, SP is not a “silver bullet.” Operating health care requires a multifaceted approach, including, among others, raising the awareness of SP services and developing community infrastructure to meet service users' needs. Furthermore, certain modifications would be needed to introduce SP in other healthcare systems, particularly in those without GPs.

This study is the first evaluation of SP from the patient's perspective through the lens of medical anthropology, by referring to the three concepts as clues for the evaluation. However, the study has limitations in design, as it is based solely on a literature review. Therefore, research by fieldwork, combining relevant criteria/clues for evaluation such as the above‐mentioned key concepts with appropriate outcome measures, appears indispensable to get a clearer picture of the impact of SP on patients' health and well‐being.

## CONFLICT OF INTEREST

The authors have stated explicitly that there are no conflicts of interest in connection with this article.
